# Cross-Disorder Pruning-Plasticity-Aging Architecture in Substance Use Disorders and Anxiety: Transcriptome-Wide Association Insights and Mechanistic Synthesis

**DOI:** 10.7759/cureus.110806

**Published:** 2026-06-13

**Authors:** Ngo Cheung

**Affiliations:** 1 Psychiatry, Cheung Ngo Medical Limited, Hong Kong, HKG

**Keywords:** addiction psychiatry, age and ageing, alcohol dependence, cannabis use disorder, clinical genomics, opioid use disorders

## Abstract

Substance use disorders and anxiety are clinically heterogeneous, and broad case-control genetic designs can obscure stage- and subtype-specific biology. A recent series of five transcriptome-wide association study (TWAS) preprints by Cheung examined opioid exposure versus dependence progression, alcohol misuse latent classes, cannabis use disorder, anxiety, and aging-related gene sets. Together, these studies suggest a cross-disorder architecture organized around a Pruning-Plasticity-Aging axis. This synthesis is narrative and hypothesis-generating, because the primary cross-disorder evidence comes from preprints and genetically predicted expression rather than measured patient expression. In this model, liability is not distributed along a single psychiatric-risk continuum. Instead, opioid exposure and some lower-risk or internalizing alcohol profiles appear to involve altered neuroimmune pruning and glial-synaptic refinement, whereas opioid dependence progression, heavier alcohol classes, broad-risk alcohol profiles, and cannabis use disorder show stronger involvement of glutamatergic plasticity, presynaptic adaptation, reward-circuit remodeling, AMP-activated protein kinase-mechanistic target of rapamycin (AMPK-mTOR) nutrient sensing, mitochondrial bioenergetics, and selected nicotinamide adenine dinucleotide (NAD)/sirtuin stress-response branches. Anxiety diverges by loading more strongly on inflammatory-apoptotic signaling, complement/sterile alpha and toll/interleukin receptor motif-containing protein 1 (SARM1)-linked axonal stress, and mitochondrial NAD-stress programmes. The most promising findings include stage-specific opposition in opioid TWAS profiles, latent-class plasticity inversion in alcohol misuse, a triggering receptor expressed on myeloid cells 2 (TREM2)-positive cannabis use disorder profile contrasted with a SARM1/complement C1q C chain (C1QC)-positive anxiety profile, and branch-specific rather than global aging biology. This review synthesizes these findings; places them in established addiction, neuroimmune, and aging biology; and outlines validation priorities for colocalization, fine-mapping, cell-type resolution, and functional testing.

## Introduction and background

Substance use disorders are conditions in which repeated substance use becomes difficult to control and causes harm, while anxiety disorders involve persistent fear, worry, or threat-related symptoms that interfere with functioning. Genetic studies can help identify inherited liability, but they often combine people at different stages of illness. Transcriptome-wide association studies (TWAS) add another layer by asking whether genetically predicted gene expression is associated with a trait. In this review, TWAS is used as a hypothesis-generating bridge between genome-wide association signals and possible brain-relevant mechanisms, not as direct evidence of gene expression changes in affected tissue.

Substance use disorders are often studied as categorical diagnoses, yet the clinical course from exposure to repeated use, dependence, withdrawal, relapse, and chronic impairment is not biologically uniform. A person with genetic liability for opioid exposure, for example, may not share the same molecular architecture as a person with liability for opioid dependence among those already exposed. Similar concerns apply to alcohol misuse, where patterns of heavy consumption, internalizing symptoms, externalizing problems, and broad functional impairment can cluster in different ways. Anxiety disorders add another layer of complexity because they often co-occur with substance use but may arise from partly different stress, inflammatory, and circuit-maintenance biology. Standard genome-wide association studies (GWAS) have helped establish the polygenic nature of substance-use and anxiety traits, but broad case-control definitions can mix exposure, opportunity, liability, progression, and severity into the same contrast [1-3].

Recent large genetic studies make the case for more careful phenotype decomposition. Polimanti et al. [3] showed that opioid use and opioid dependence should not be treated as interchangeable genetic outcomes. Thijssen et al. [4] used genetically informed latent-class approaches to examine alcohol misuse subgroups, distinguishing heavy-use and broad-risk profiles rather than reducing alcohol misuse to one severity scale. Levey et al. [5] expanded the genetic evidence base for cannabis use disorder across ancestries, while Strom et al. [6] identified a large set of loci for major anxiety disorders and highlighted GABAergic signaling. These studies illustrate a broader point: psychiatric genetics increasingly needs stage-aware and subtype-aware frameworks, especially for disorders in which exposure, repeated reinforcement, withdrawal, negative affect, and comorbidity are all relevant.

TWAS provide one way to connect genome-wide association results to tissue-relevant gene-expression mechanisms. Methods such as PrediXcan and related summary-statistic TWAS approaches use genetically regulated expression models to test whether predicted expression of a gene is associated with a phenotype [7,8]. Multi-tissue and tissue-specific approaches have been extended through GTEx-based resources and S-PrediXcan-type frameworks, allowing researchers to examine brain regions that are plausibly relevant to reward, habit, stress, and affective regulation [9,10]. TWAS is not a direct measure of expression in patient tissue, and it does not by itself prove causality, but it can sharpen hypotheses about the genes and pathways most likely to mediate genetic liability [11,12].

A second reason to revisit substance-use and anxiety genetics is that many of the implicated pathways overlap with cellular maintenance and aging biology. Aging is not a single process. It includes altered proteostasis, mitochondrial dysfunction, nutrient sensing, cellular senescence, genomic instability, telomere maintenance, inflammatory output, and intercellular communication [13,14]. Nicotinamide adenine dinucleotide (NAD) metabolism, sirtuin signaling, mitochondrial stress, and DNA repair can influence neuronal resilience and glial function across the lifespan [15-17]. These pathways are relevant to addiction because chronic drug exposure and withdrawal place stress on synapses, mitochondria, glia, and intracellular signaling systems. They are also relevant to anxiety because inflammatory signaling, apoptosis thresholds, and stress-sensitive mitochondrial pathways have long been linked to affective symptoms and sickness behavior [18,19].

Several terms are used throughout this review in a specific way. Pruning refers to synapse or neurite refinement processes in which complement, microglia, astrocytes, and related signals may influence which connections are maintained or removed. Microglial remodeling refers more narrowly to immune-cell state changes, phagocytosis, lipid handling, and synapse-related surveillance, whereas axonal degeneration refers to injury-like axon-loss programs such as sterile alpha and toll/interleukin receptor motif-containing protein 1 (SARM1)-linked NAD depletion. Plasticity refers to changes in synaptic strength, receptor composition, presynaptic release, and circuit learning. Aging-related biology is used here for cellular maintenance pathways such as NAD/sirtuin signaling, mitochondrial stress, telomere and DNA-damage response, senescence-related gene sets, and apoptosis thresholds; it does not mean that a TWAS signal proves accelerated chronological aging.

The neurobiology of addiction already points toward several modules that are likely to intersect with aging-related pathways. Glutamate homeostasis in the nucleus accumbens is central to drug seeking, relapse, and impaired behavioral flexibility [20-22]. Drug-evoked synaptic plasticity changes corticostriatal and limbic circuits over time [22]. Habit formation and compulsive drug seeking depend on a gradual shift from ventral striatal reward learning to more dorsal striatal control [23]. The transition from reward-driven use to negative reinforcement also involves anti-reward and stress systems, including dynorphin and kappa-opioid receptor signaling [24,25]. These mechanisms do not operate separately from cellular maintenance. Synapses that are potentiated, weakened, pruned, or remodeled also require energy, protein turnover, mitochondrial support, and glial surveillance.

The five Cheung preprints build on this background by examining TWAS profiles across opioid, alcohol, cannabis use disorder, and anxiety phenotypes against aging, pruning, glutamatergic, metabolic, NAD/sirtuin, senescence, and apoptosis-related gene sets. Across these papers, the strongest emerging idea is not that substance use disorders and anxiety show one shared “accelerated aging” program. Instead, the evidence supports a more specific Pruning-Plasticity-Aging architecture. In this framework, genetic liability is distributed across partially antagonistic molecular modules: neuroimmune pruning and synaptic refinement, glutamatergic and reward-circuit plasticity, bioenergetic and mitochondrial adaptation, NAD/sirtuin and DNA-repair routing, senescence-like stress adaptation, inflammatory-apoptotic signaling, axonal stress, and presynaptic G protein-coupled receptor (GPCR)/cyclic adenosine monophosphate (cAMP)/calcium adaptation [26-30].

This review synthesizes these five TWAS preprints and the derived cross-disorder model. The aim is not to present the findings as validated mechanisms. Rather, it is to organize the most promising signals into a coherent, testable framework. The review emphasizes stage-specific opposition in opioid genetics, latent-class plasticity inversion in alcohol misuse, triggering receptor expressed on myeloid cells 2 (TREM2)/SARM1 divergence between cannabis use disorder and anxiety, branch-specific NAD/sirtuin biology, and translational implications for stage- and subtype-matched precision psychiatry. A translational thread is retained only as a non-clinical research hypothesis mapping for future biomarker and experimental studies. No clinical utility is established, no biomarker panel is validated, and no prospective stratified trial currently supports treatment matching from these TWAS profiles.

## Review

Synthesis methods

This article is a narrative mechanistic review and hypothesis synthesis. It is not a systematic review, does not claim Preferred Reporting Items for Systematic Reviews and Meta-Analyses (PRISMA)-level reproducibility, and does not perform a new meta-analysis, meta-regression, or risk-of-bias assessment. The five Cheung preprints are treated as the primary source material because they generated the cross-disorder TWAS patterns being synthesized. External sources are used to test whether the proposed modules are biologically plausible, independently supported, or contradicted by established genetic, neurobiological, aging, and model-system literature.

This synthesis is based on five Cheung TWAS preprints: one examining opposing aging-related transcriptomic profiles in opioid exposure versus dependence liability; one examining aging pathways in alcohol misuse and anxiety; one examining cannabis use disorder and anxiety GWAS signals against aging gene sets; one examining latent-class TWAS profiles in alcohol misuse subtypes; and one examining opioid exposure versus dependence progression using stage-specific transcriptomic architecture [26-31]. These preprints serve as the primary source material for the proposed cross-disorder framework. The review also integrates established methodological and mechanistic literature on TWAS, addiction neurobiology, complement-mediated pruning, TREM2 and SARM1 biology, glutamatergic plasticity, AMP-activated protein kinase-mechanistic target of rapamycin (AMPK-mTOR) signaling, mitochondrial metabolism, NAD/sirtuin pathways, senescence, apoptosis, and precision treatment development.

Supporting literature was selected narratively from fields directly relevant to the modules under discussion: GWAS phenotype decomposition, TWAS methodology, opioid and alcohol addiction neurobiology, glutamate homeostasis, complement-mediated synaptic refinement, TREM2 and SARM1 biology, NAD/sirtuin and senescence biology, AMPK-mTOR nutrient sensing, apoptosis, and early glucagon-like peptide-1 (GLP-1) receptor agonist work in substance use disorders (Table 1). Because the synthesis was not conducted as a systematic search, absence of a cited study should not be interpreted as evidence that no such study exists.

**Table 1:** Source phenotype and analytic context carried forward in this synthesis. TWAS = transcriptome-wide association study; CUD = cannabis use disorder; GLP-1 = glucagon-like peptide-1; KATP = ATP-sensitive potassium channel; AMPK-mTOR = AMP-activated protein kinase-mechanistic target of rapamycin

Major claim in this synthesis	Cheung preprint evidence	Independent external evidence	Contradictions or competing explanations
Opioid exposure and dependence progression should be separated	Stage-specific opioid TWAS profiles and reported sign-sensitive genes [1,2]	Opioid use and opioid dependence differ genetically in the PGC study [3]. KOR/dynorphin and opioid cellular-adaptation literature supports dependence-stage negative-affect and intracellular-adaptation models [4-7]	Exposure can reflect prescribing, pain, access, and medical context. Dependence signals may also reflect chronic pain, psychiatric comorbidity, or ascertainment differences
Alcohol misuse latent classes may have different plasticity profiles rather than one severity continuum	Class 2 versus Class 3/Class 4 glutamatergic-plasticity opposition and Class 4 neuroimmune-pruning signals [8,9]	Latent-class alcohol misuse structure is supported by genetically informed alcohol-misuse work [10]. Glutamate homeostasis, corticostriatal plasticity, and habit circuitry are established in addiction biology [11-14]. Adenosine A2A and dopamine D2 interactions support striatal reward-habit interpretation [15-17]	Class labels may not map cleanly onto clinical phenotypes. Gene-set opposition may be influenced by tissue models, gene-set overlap, or a few high-influence genes
Pruning-related biology differs between CUD and anxiety	TREM2-positive CUD profile contrasted with SARM1/C1QC-positive anxiety profile [18]	TREM2 is linked to microglial lipid handling and disease-associated microglial states [19,20]. SARM1 is linked to NAD cleavage and programmed axon degeneration [21-23]. Complement participates in synapse elimination and microglial pruning [24-26]	The contrast may reflect different GWAS designs, ancestry composition, tissue prediction performance, or MHC/complement linkage disequilibrium rather than disorder-specific pruning biology
Aging biology is branch-specific rather than globally accelerated	Different NAD/sirtuin, senescence-like, mitochondrial, and apoptosis branches across opioid, alcohol, CUD, and anxiety contrasts [1,2,8,9,18]	Aging hallmarks, NAD metabolism, sirtuin biology, senescence, and neuronal cell-death literature support the biological separability of these branches [27-38]	Aging gene sets overlap heavily with general stress-response genes. TWAS cannot distinguish adaptive compensation from vulnerability without measured expression and longitudinal data
GLP-1, KATP, AMPK-mTOR, and mitochondrial genes may connect metabolic state to reward liability	CUD reward-metabolic branch involving GLP1R, PCSK2, KCNJ11, ABCC8, AMPK-mTOR, and cAMP/calcium-related genes [18]	KATP channels couple cellular energy state to excitability [39]. AMPK and mTOR are central nutrient and energy sensors [40,41]. A systematic review of GLP-1 receptor agonists in substance-use disorders found strong preclinical evidence across substances but preliminary, heterogeneous clinical evidence mainly in alcohol and nicotine contexts [42]	GLP-1 receptor agonist evidence is not treatment-selection evidence for CUD. Clinical data remain limited, short-term, and not based on TWAS-defined molecular subgroups

The synthesis approach was qualitative and mechanism-focused. Findings were prioritized when they showed recurring support across more than one analytic contrast or more than one disorder, when they were supported by multiple metrics within the source papers, when influential genes were identified through leave-one-out or sign-flip analyses, and when the gene-level interpretation was biologically plausible. The most important analytic features carried forward from the source papers were Stouffer Z enrichment, Wilcoxon-type tests, profile correlations, sign-concordance estimates, cosine similarity, leave-one-out influence, and directional sign-flip patterns. Particular weight was given to findings in which the same pathway domain showed opposite direction across phenotype definitions, because this pattern is less consistent with a simple severity model and more consistent with stage- or subtype-specific biology.

For gene prioritization, Tier 1 genes were defined as genes that met several of the following criteria in the source synthesis: recurrence across more than one contrast or disorder, centrality within an anchor pathway, contribution to a stage- or subtype-discriminating pattern, biological support from independent literature, and practical testability in cell-type or circuit models. Tier 2 genes were defined as genes with supportive but less recurrent, less discriminating, or more context-dependent evidence. This rubric is intended for prioritizing validation, not for ranking causal certainty. Genes in MHC/complement regions, genes from small gene sets, and genes with strong linkage disequilibrium risk require especially cautious interpretation.

The TWAS framework used in the source papers relied on genetically predicted expression rather than measured patient expression. As in other TWAS applications, the findings should be read as associations between inherited regulatory variation and phenotype liability, not as direct claims of upregulation or downregulation in diseased tissue [7,8,11]. The use of GTEx v8 brain-tissue prediction models and S-PrediXcan-style analyses gives the results tissue relevance, but it does not fully resolve cell type, developmental timing, or causal direction [9,10,12]. The source papers also used curated aging-related and pathway-focused gene sets. Gene-set approaches can clarify higher-order biology, but small gene sets and overlapping gene membership can make interpretation unstable. For these reasons, all findings are treated as hypothesis-generating until they are supported by colocalization, fine-mapping, replication, measured expression, proteomic validation, and functional experiments.

Independent external evidence

Because the core synthesis depends on five related preprints, independent evidence is essential for evaluating whether the proposed modules are plausible (Table 2). The external literature does not validate the Cheung architecture as a whole, but it does support several component mechanisms: opioid exposure and dependence are genetically separable; glutamate homeostasis is central to addiction; complement and microglia participate in synaptic refinement; TREM2 and SARM1 mark different immune-remodeling and axonal-degeneration biology; NAD/sirtuin, mitochondrial, senescence, and apoptosis pathways are central to cellular maintenance; and GLP-1 receptor agonists are being investigated in substance use disorders. The same literature also highlights major uncertainties, including linkage disequilibrium, cell-type mixture, tissue mismatch, reverse biological interpretation, and lack of causal evidence.

**Table 2:** Major claims, external support, and competing explanations. TWAS = transcriptome-wide association study; TREM2 = triggering receptor expressed on myeloid cells 2; CUD = cannabis use disorder; SARM1 = sterile alpha and toll/interleukin receptor motif-containing protein 1; NAD = nicotinamide adenine dinucleotide; GWAS = genome-wide association studies

Major claim in this synthesis	Cheung preprint evidence	Independent external evidence	Contradictions or competing explanations
Opioid exposure and dependence progression should be separated	Stage-specific opioid TWAS profiles and reported sign-sensitive genes	Opioid use and opioid dependence differ genetically in the PGC study. KOR/dynorphin and opioid cellular adaptation literature supports dependence-stage negative-affect and intracellular adaptation models	Exposure can reflect prescribing, pain, access, and medical context. Dependence signals may also reflect chronic pain, psychiatric comorbidity, or ascertainment differences
Alcohol misuse latent classes may have different plasticity profiles rather than one severity continuum	Class 2 versus Class 3/Class 4 glutamatergic-plasticity opposition and Class 4 neuroimmune-pruning signals	Latent-class alcohol misuse structure is supported by Thijssen et al. [4]. Glutamate homeostasis, corticostriatal plasticity, and habit circuitry are established in addiction biology. Adenosine A2A and dopamine D2 interactions support striatal reward-habit interpretation	Class labels may not map cleanly onto clinical phenotypes. Gene-set opposition may be influenced by tissue models, gene-set overlap, or a few high-influence genes
Pruning-related biology differs between CUD and anxiety	TREM2-positive CUD profile contrasted with SARM1/C1QC-positive anxiety profile	TREM2 is linked to microglial lipid handling and disease-associated microglial states. SARM1 is linked to NAD cleavage and programmed axon degeneration. Complement participates in synapse elimination and microglial pruning	The contrast may reflect different GWAS designs, ancestry composition, tissue prediction performance, or MHC/complement linkage disequilibrium rather than disorder-specific pruning biology
Aging biology is branch-specific rather than globally accelerated	Different NAD/sirtuin, senescence-like, mitochondrial, and apoptosis branches across opioid, alcohol, CUD, and anxiety contrasts	Aging hallmarks, NAD metabolism, sirtuin biology, senescence, and neuronal cell-death literature support the biological separability of these branches	Aging gene sets overlap heavily with general stress-response genes. TWAS cannot distinguish adaptive compensation from vulnerability without measured expression and longitudinal data
GLP-1, KATP, AMPK-mTOR, and mitochondrial genes may connect metabolic state to reward liability	CUD reward-metabolic branch involving GLP1R, PCSK2, KCNJ11, ABCC8, AMPK-mTOR, and cAMP/calcium-related genes	KATP channels couple cellular energy state to excitability. AMPK and mTOR are central nutrient and energy sensors. A systematic review of GLP-1 receptor agonists in substance-use disorders found strong preclinical evidence across substances but preliminary, heterogeneous clinical evidence mainly in alcohol and nicotine contexts	GLP-1 receptor agonist evidence is not treatment-selection evidence for CUD. Clinical data remain limited, short-term, and not based on TWAS-defined molecular subgroups

Opioid stage-specific architecture: exposure versus dependence progression

The opioid papers provide one of the clearest examples of why phenotype definition matters. Opioid exposure liability and opioid dependence progression do not appear to represent weak and strong versions of the same transcriptomic signal. In the Cheung opioid analyses, exposure-related contrasts were reported as predicted-expression association patterns consistent with altered complement and pruning signals, while dependence-among-exposed contrasts shifted toward negative-affect, cAMP/calcium adaptation, pain persistence, mitochondrial metabolism, AMPK-mTOR signaling, and selected NAD/sirtuin branches [27,29]. This is consistent with the broader genetic argument that opioid exposure and opioid dependence are separable phenotypes that should be analyzed separately when possible [3].

The exposure side of the architecture was most closely tied to altered neuroimmune pruning and glial-synaptic refinement. The relevant genes include *C1QA*, *C1QB*, *C1QC*, *C4A*, *CR1*, *CD46*, *CD59*, *CFB*, and *CFI*, together with astrocyte and glutamate-homeostasis genes such as *GRM3*, *SLC1A3*, and *ATP1A2*. The interpretation is not that reduced complement signaling alone causes opioid exposure. Exposure is partly shaped by pain indication, prescribing context, availability, and social environment. The TWAS pattern is more limited and more useful: inherited regulatory differences linked to exposure appear to involve glial support, complement-mediated synaptic tagging, astrocytic glutamate regulation, and early neuroactive response systems. *HTR1B* and *ADCY6* also appear in this stage-sensitive context, suggesting that serotonergic and cAMP-linked signaling may contribute to early reward, impulsivity, or exposure-related liability in ways that differ from dependence progression [27,29].

Dependence progression, by contrast, shows predicted-expression enrichment for intracellular adaptation under repeated opioid stress. *OPRK1* is a central gene in this interpretation because the kappa-opioid receptor and dynorphin system are strongly tied to dysphoria, stress-induced drug seeking, and negative reinforcement [24,31,32]. The opioid dependence profile also includes *KCNIP3*, also known as *DREAM*, along with *CREM*, *FOS*, *PRKAR2A*, *PRKAR2B*, *PRKACB*, *ADCY6*, *ADCY9*, *PDE4B*, *PDE1B*, *PDE4A*, *PPP3R1*, *PPP3CA*, *PPP3CC*, *PLCB3*, *GNG10*, *GNG12*, *GNAQ*, *GNAI3*, and *GNAL*. This gene cluster points toward cAMP rebound, protein kinase A (PKA) and phosphodiesterase (PDE) remodeling, GPCR-linked calcium signaling, calcineurin pathways, and activity-dependent transcription. These processes fit long-standing evidence that opioid dependence involves cellular and synaptic adaptations, not only acute receptor activation [33].

One of the strongest stage-specific observations is the reported *GCH1* sign-flip. In the source synthesis, *GCH1* is negative in exposure-related analyses but positive in dependence-among-exposed contrasts. Here, negative and positive refer only to TWAS predicted-expression association direction, not to measured expression in patient tissue. *GCH1* encodes GTP cyclohydrolase 1, a key enzyme in tetrahydrobiopterin biology, and tetrahydrobiopterin is relevant to pain sensitivity and persistence [34]. The sign-flip supports a two-stage pain model. Pain biology may influence opioid receipt or exposure, while persistent pain, hyperalgesia, affective pain amplification, or monoaminergic/nitric oxide compensation may become more relevant to dependence progression. Other pain-linked genes in the broader module include *TRPV1*, *TAC1*, *TACR1*, *P2RX2*, *P2RX4*, *P2RX7*, *NTRK1*, *SCN11A*, and *EDNRB*. This does not prove that these genes cause opioid dependence, but it gives a concrete route for testing whether pain-stage biology helps distinguish exposure from progression.

The dependence profile also contains strong metabolic and aging-related signals. *LAMTOR5*, together with *LAMTOR3*, *LAMTOR4*, *SLC38A9*, *MTOR*, *TSC1*, *RHEB*, and *RPTOR*, places lysosomal nutrient sensing and mTORC1 recruitment near the center of dependence-stage biology. This is biologically plausible because the Ragulator-Rag complex links amino-acid sensing to mTORC1 localization and activation at the lysosome [35,36]. *DLD* is another important dependence-linked gene. It encodes dihydrolipoamide dehydrogenase, a component shared by major mitochondrial dehydrogenase complexes, including pyruvate dehydrogenase and alpha-ketoglutarate dehydrogenase complexes [37]. Its recurrence suggests that dependence liability may involve mitochondrial flux, NADH production, and oxidative metabolism. Additional mitochondrial and metabolic genes in the architecture include *OGDH*,* PDHB*,* DLST*,* NDUFS1*,* NDUFC2*,* COX5A*,* CYC1*, and related oxidative phosphorylation components.

The opioid dependence findings also connect to branch-specific NAD and sirtuin biology. *SIRT6* appears as a recurring regulatory hub, and *NAMPT* is described as context-dependent or sign-flipping across contrasts. *SIRT6* is involved in chromatin regulation, telomere biology, DNA repair, inflammatory repression, and metabolic stress responses [15,38]. In the opioid setting, *SIRT6*,* NAMPT*,* NT5E*,* DLD*,* and mTOR*-related genes together point toward a stress-adaptation branch rather than a simple accelerated-aging signature. *STX1A* and other presynaptic genes add a synaptic-release layer, suggesting that dependence progression may involve the combined remodeling of presynaptic function, intracellular second-messenger pathways, pain biology, mitochondrial state, and chromatin-linked stress resilience [27,29].

Alcohol latent-class glutamatergic plasticity and neuroimmune architecture

The alcohol latent-class analyses provide the strongest subtype evidence in the Cheung series. Rather than treating alcohol misuse as one continuum, the source papers build on latent-class work that separates lower-risk or internalizing-light profiles from heavier and broader-risk classes [4]. The key finding is a reported inversion or anti-correlation in glutamatergic and synaptic-plasticity profiles between Class 2 and higher-risk classes, especially Class 3 and Class 4 [26]. This statement refers to profile-level TWAS direction and similarity metrics described in the source preprint; the present synthesis does not re-estimate the correlation coefficients, confidence intervals, or null distributions. This is important because it means that a lower-consumption or internalizing-linked class should not be treated automatically as a simple control state. It may have its own transcriptomic architecture.

Class 2 appears to carry a plasticity profile that is directionally opposed to heavier-use profiles. The interpretation is cautious: Class 2 may reflect internalizing-linked alcohol behavior, reduced consumption, non-drinking, or different cortical-limbic glutamatergic set points. It should not be overread as resilience or pathology without behavioral and clinical validation. Still, the anti-correlation itself matters because it shows that alcohol misuse subtypes may differ not only in severity but in the direction of synaptic and glutamatergic liability. This fits broader addiction evidence that glutamate signaling and corticostriatal plasticity are central to drug seeking and habit formation, but it adds the point that these systems may be organized differently across alcohol subgroups [20-22].

Class 3 is closer to a heavy-use reward and habit profile. Genes emphasized in this module include *ADORA2A*,* DRD2*,* PPP1R1B*,* COMT*,* GRM3*,* STX1A*,* VAMP2*,* SNAP25*,* and CLN3*. *ADORA2A* is especially notable because adenosine A2A receptors interact functionally with dopamine D2 receptor signaling in the striatum, shaping dopamine-glutamate integration and corticostriatal plasticity thresholds [39-41]. *DRD2* and *PPP1R1B*, which encode DARPP-32, add a dopamine and cAMP integration layer, while *STX1A*,* VAMP2*,and* SNAP25* point toward presynaptic release. Together, the Class 3 pattern is compatible with heavy-use reinforcement learning, cue-related plasticity, and early habit formation.

Class 4 appears broader and more clinically impaired. In the Cheung synthesis, Class 4 adds neuroimmune pruning and circuit-maintenance vulnerability on top of the reward-habit plasticity profile [26,28]. Genes in this branch include *CLN3*,* ADORA2A*,* TREM2*,* C1QA*,* C1QB*,* C1QC*,* C3*,* CX3CR1*,* APOE*,* DRD2*,* PPP1R1B*,* COMT*,* STX1A*,* VAMP2*,* SNAP25*, and* GRM3*. *TREM2* is relevant because it is involved in microglial lipid sensing, phagocytosis, and disease-associated microglial state changes [42,43]. Complement genes such as *C1QA*,* C1QB*,* C1QC*,* C3*, and* C4A* are relevant because classical complement signaling can tag synapses for microglial elimination [44-46]. The alcohol Class 4 profile therefore suggests a state in which heavy-use plasticity is accompanied by vulnerability in synaptic maintenance, microglial remodeling, and pruning-related circuit refinement.

*CLN3* is one of the most interesting alcohol-linked genes because it connects synaptic plasticity to lysosomal-autophagic maintenance. *CLN3* is best known in the context of Batten disease biology, but its broader relevance includes lysosomal function, autophagy, membrane trafficking, and neuronal maintenance [47]. In this framework, *CLN3* is not simply a disease gene imported into alcohol biology. It acts as a possible bridge between plasticity and maintenance. A circuit undergoing repeated alcohol-related remodeling may require efficient synaptic protein turnover, lysosomal handling, and mitochondrial quality control. If that maintenance capacity is genetically altered, heavy-use biology may become more likely to progress toward broad impairment.

The aging-focused alcohol paper adds a caudate-centered senescence-like stress program [28]. More precisely, this refers to predicted-expression enrichment in senescence-related gene-set members involved in DNA-damage response, telomere maintenance, checkpoint signaling, and chromatin regulation, not evidence of measured senescent-cell burden in caudate tissue. The relevant genes include *YPEL3*,* SIRT6*,* WRN*,* TERF2*,* POT1*,* TP53*,* ATM*,* CDKN1A*,* CDKN1B*,* PML*,* ULK3*,* ABL1*,* RFC1*, and* CHTF8*. The caudate is part of dorsal striatal habit circuitry, and dorsal striatal mechanisms are central to the shift from flexible action to compulsive responding [23]. The alcohol senescence-like signature should not be taken as proof that senescent cells are accumulating in alcohol-related brain tissue. A more careful interpretation is that inherited regulation of DNA damage response, telomere maintenance, chromatin control, checkpoint signaling, and stress adaptation is enriched in alcohol subtype contrasts involving dorsal striatal circuitry. This is consistent with modern aging biology, which treats senescence-related genes as part of a broader stress-response and tissue-maintenance network rather than a single binary cell state [48-50].

Cannabis use disorder versus anxiety divergence against aging gene sets

The cannabis use disorder and anxiety comparison is important because it shows that shared pathway labels can hide sharply different gene-level architecture. Cannabis use disorder and anxiety show some overlap in glial-metabolic vulnerability, including adult oligodendrocyte and PI3K-AKT-mTOR-related signals [30]. Genes in this shared background include *UGT8*,* CLDN11*,* ENPP2*,* GPR37*,* GPNMB*,* FOLH1*,* GLYCTK*,and *PLEKHB1*. This shared layer may reflect myelin, lipid metabolism, glial stress, or broader brain-resilience biology rather than a disorder-specific mechanism.

The clearest divergence is in pruning-related biology. The cannabis use disorder profile is described as TREM2-positive, with additional involvement of NGEF, ADGRB3, ITGB1, VANGL2, and CX3CR1 in selected outputs. This suggests microglial remodeling, dendritic or synaptic structural adaptation, adhesion-related engulfment, and neuron-microglia communication. In contrast, the anxiety profile is SARM1-positive and C1QC-positive, with additional EPHA4, PLXNC1, C4A, and C1Q-family involvement [30]. SARM1 is a central mediator of programmed axon degeneration because its TIR domain has intrinsic NAD-cleavage activity, linking axonal injury to NAD depletion [51-53]. The contrast between TREM2-positive cannabis use disorder and SARM1/C1QC-positive anxiety is therefore not just a difference in gene names. It suggests distinct pruning states: microglial clearance and remodeling in cannabis use disorder versus axonal-stress and complement-linked pruning in anxiety.

The cannabis use disorder side also shows a reward-metabolic plasticity cascade. In TWAS-appropriate terms, this means predicted-expression associations in genes related to reward circuitry, glutamatergic signaling, vesicle release, dopamine-cAMP integration, and metabolic nutrient sensing. In the nucleus accumbens and related reward circuitry, the relevant genes include *GRM3*,* GRIN1*,* GRIA2*,* GRIA4*,* HOMER1*,* DLG4*,* STX1A*,* VAMP2*,* STXBP1*,* PPP1R1B*,* COMT*,* DRD2*,* PDE4B*,* PDE4C*, and *PDE4D*. These genes connect glutamate receptor signaling, postsynaptic scaffolding, vesicle release, dopamine-cAMP integration, and relapse-relevant plasticity. This fits the established role of glutamate homeostasis in addiction and the nucleus accumbens as a cross-drug site where glutamatergic dysregulation contributes to drug seeking [20,21]. The cannabis use disorder TWAS architecture extends that model by adding a metabolic layer.

That metabolic layer includes *GLP1R*,* PCSK2*,* KCNJ11*,* ABCC8*,* PRKAA1*,* STK11*,* RPTOR*,* TSC1*,* PRKAG1*,* ADCY6*,* PRKACA*,* RAPGEF3*,* PLCB3*,* CACNA1C*,* CACNA1D*, and* CACNA1E*. *KCNJ11* and *ABCC8* encode components of ATP-sensitive potassium channels, which couple cellular energy state to excitability [54]. *GLP1R* and *PCSK2* point toward incretin, insulin-related, and metabolic-reward biology. AMPK-related genes, including *PRKAA1*, *STK11*, and *PRKAG1*, connect energy stress to cellular adaptation [55]. mTOR-related genes add nutrient-sensing and plasticity-relevant protein synthesis [56]. Because GLP-1 receptor agonists are now being investigated as potential modulators of substance use behavior, these cannabis use disorder findings are translationally interesting, although still far from treatment-selection evidence [57]. A recent systematic review of GLP-1 receptor agonists in substance use disorders reported 42 included primary studies, with 36 preclinical and six clinical investigations. The review found consistent preclinical support across several substances but described clinical evidence as preliminary, heterogeneous, and strongest so far in alcohol and nicotine contexts rather than as a validated treatment-matching strategy [57].

Anxiety, in contrast, is weighted more strongly toward inflammatory, apoptotic, and NAD-stress biology. This weighting should be read as gene-set enrichment and predicted-expression association, not proof of active inflammatory or apoptotic pathology in patient tissue. The inflammatory module includes *TNF*,* NFKB1*,* RELA*,* TLR4*,* TLR9*,* MYD88*,* IRAK4*,* PYCARD*,* IL1B*,* NOS2*,* STAT1*, and* CHUK*. These genes point toward innate immune danger sensing, NF-kappa-B signaling, inflammasome activity, and cytokine amplification. The apoptosis and mitochondrial death-threshold module includes *BAK1*,* BAD*,* BIK*,* BCL2L1*,* BCL2*,* MCL1*,* CASP10*,* CASP7*,* MGMT*,* NEK4*,* NEK6*,* OPA1*,* PNPT1*, and* MAPK14*. These genes are consistent with known intrinsic apoptosis and mitochondrial stress pathways [58,59]. The NAD-stress branch includes *SARM1*,* SIRT3*,* SIRT5*,* NADK2*,* NMRK1*,* NAMPT*,* NT5C3A*,* NAXD*,* HADH*,* TNKS*, and* TNKS2*. The overall anxiety profile is therefore better described as inflammatory-apoptotic-axonal NAD stress than as generalized aging.

Integrated cross-disorder Pruning-Plasticity-Aging architecture

**Figure 1:**
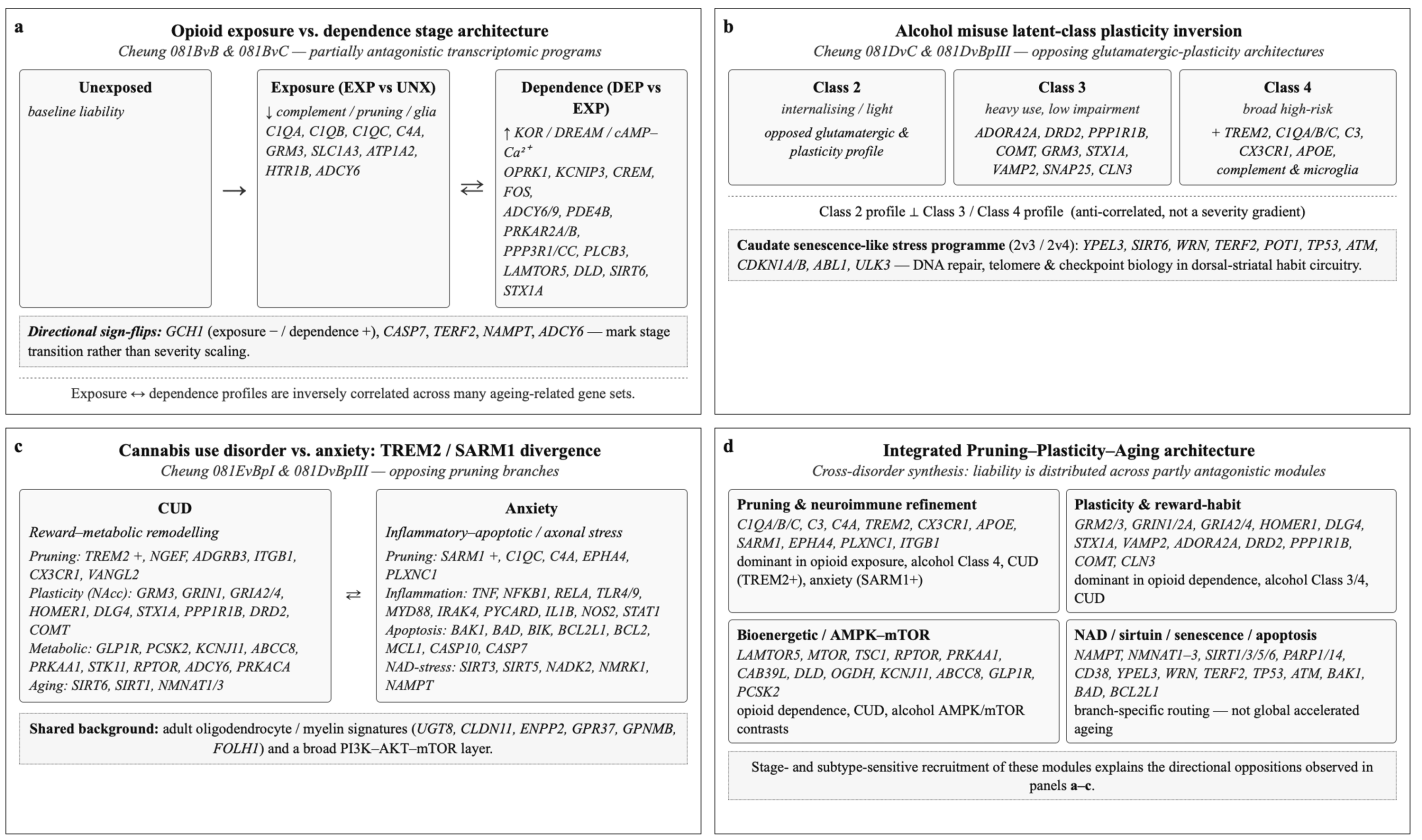
Schematic synthesis of the five Cheung TWAS preprints addressed in the Results section. (a) Opioid exposure liability and opioid dependence progression engage partially antagonistic transcriptomic programmes: complement/glial-refinement signals on the exposure side versus KOR–DREAM, cAMP–Ca²⁺, mitochondrial and mTOR/NAD adaptation on the dependence side, with gene-level sign-flips (e.g., *GCH1*,* CASP7*,* TERF2*,* NAMPT*,* ADCY6*) marking stage transition. (b) In genetically stratified alcohol-misuse latent classes, the Class 2 profile is directionally opposed to Class 3/Class 4 across glutamatergic and synaptic-plasticity gene sets; Class 4 adds neuroimmune-pruning and complement involvement, and a caudate-centered senescence-like stress program appears in 2v3/2v4 contrasts. c, Cannabis use disorder and anxiety share a glial-metabolic background (oligodendrocyte/myelin, PI3K–AKT–mTOR) but diverge sharply on the pruning axis, with a TREM2-positive microglial-remodelling profile in CUD and a SARM1/C1QC-positive axonal-stress and complement profile in anxiety; CUD additionally loads on a nucleus accumbens glutamatergic and GLP1/insulin/KATP/AMPK reward-metabolic cascade, whereas anxiety loads on TNF–NFκB cytokine, intrinsic apoptosis and mitochondrial NAD-stress biology. (d) The integrated Pruning–Plasticity–Aging architecture organizes these findings into four interacting modules whose stage- and subtype-specific recruitment, rather than a single severity continuum, accounts for the cross-disorder pattern. All gene assignments are statistical associations between genetically predicted expression and trait liability and require colocalization, fine-mapping and functional validation. EXP = opioid exposure liability; DEP|EXP = opioid dependence among exposed or dependence progression; CUD = cannabis use disorder; NAcc = nucleus accumbens; KATP = ATP-sensitive potassium channel; GLP-1 = glucagon-like peptide-1; AMPK-mTOR = AMP-activated protein kinase-mechanistic target of rapamycin

Across the five preprints, the most useful synthesis is a Pruning-Plasticity-Aging architecture with several linked but distinguishable poles (Figure 1). The pruning and neuroimmune refinement pole includes complement-mediated synaptic tagging, microglial engulfment, astrocyte-microglia crosstalk, MHC-related immune regulation, and axonal degeneration-related pruning. The recurring genes include *C1QA*,* C1QB*,* C1QC*,* C3*,* C4A*,* TREM2*,* CX3CR1*,* APOE*,* SARM1*,* ITGB1*,* ADGRB3*,* EPHA4*,* PLXNC1*,* VANGL2*, and* NGEF*. This pole is not uniformly increased or decreased across disorders. Opioid exposure shows reduced or altered complement/pruning signal; alcohol Class 4 shows broader TREM2, complement, and circuit-maintenance involvement; cannabis use disorder shows TREM2-positive remodeling; and anxiety shows SARM1/C1QC-positive axonal-stress pruning [26-30].

The plasticity and reward-habit pole includes glutamatergic signaling, corticostriatal learning, long-term potentiation and depression, cue reactivity, habit formation, synaptic scaffolding, and presynaptic vesicle release. Key genes include *GRM2*,* GRM3*,* GRIN1*,* GRIN2A*,* GRIA2*,* GRIA4*,* GRIK2*,* HOMER1*,* DLG4*,* ARC*,* DBN1*,* FXR1*,* NCSTN*,* GSK3B*,* STX1A*,* VAMP2*,* STXBP1*,* SNAP25*,* CLN3*,* ADORA2A*,* DRD2*,* PPP1R1B*, and* COMT*. This module is strongest in opioid dependence progression, alcohol heavy-use and broad-risk classes, and cannabis use disorder reward circuitry. It is also the module most directly related to glutamatergic homeostasis and relapse biology [20-22].

The bioenergetic and AMPK-mTOR pole includes cellular energy sensing, mitochondrial oxidative metabolism, TCA-cycle flux, nutrient sensing, lysosomal mTOR recruitment, autophagy balance, and ATP-state-to-excitability coupling. The key genes include *LAMTOR5*,* LAMTOR3*,* LAMTOR4*,* SLC38A9*,* MTOR*,* TSC1*,* TSC2*,* RHEB*,* RPTOR*,* PRKAA1*,* PRKAB1*,* PRKAG1*,* CAB39L*,* STK11*,* DLD*,* OGDH*,* PDHB*,* DLST*,* NDUFS1*,* NDUFS3*,* NDUFC2*,* COX5A*,* COX6B1*,* CYC1*,* TFAM*,* VDAC2*,* MTCH2*,* CLN3*,* KCNJ11*,* ABCC8*,* GLP1R*, and* PCSK2*. This pole is prominent in opioid dependence and cannabis use disorder, and it appears in alcohol subtype contrasts through AMPK-mTOR and stress-adaptation genes. Anxiety contains mitochondrial stress biology too, but its signal is more weighted toward inflammatory-apoptotic and NAD-depletion pathways than toward adaptive GLP1/KATP/AMPK reward-metabolic remodeling.

The NAD, sirtuin, senescence, and apoptosis pole is the most nuanced. It includes NAD salvage and synthesis genes such as *NAMPT*,* NAPRT*,* NMRK1*,* NMNAT1*,* NMNAT2*,* NMNAT3*,* NADK2*,* NAXD*, and* QPRT*;sirtuins such as SIRT1, SIRT2, SIRT3, SIRT5, and SIRT6; NAD-consuming enzymes such as PARP1, PARP14, PARP15, CD38, TNKS, and TNKS2; senescence and DNA-repair genes such as *YPEL3*,* WRN*,* TERF2*,* POT1*,* TP53*,* ATM*,* CDKN1A*,* CDKN1B*,* PML*,* ABL1*,* ULK3*,* MORC3*,* TBX2*,* ZMPSTE24*,* MAPKAPK3*, and* MAPKAPK5*; and apoptosis genes such as *BAK1*,* BAD*,* BIK*,* BCL2L1*,* BCL2*,* MCL1*,* BCL2L11*,* CASP7*, and* CASP10*. The central point is that aging biology is redistributed by branch. Opioid dependence and cannabis use disorder show more SIRT6/SIRT1 and metabolic-regulatory routing; alcohol subtypes show caudate senescence-like stress and DNA-repair programmes; anxiety shows SARM1, mitochondrial sirtuins, inflammatory signaling, and apoptosis thresholds.

Taken together, the model can be stated plainly. Genetic liability for opioid exposure and certain lower-risk or internalizing alcohol profiles tends to involve altered neuroimmune pruning and glial-synaptic refinement. Opioid dependence progression, heavier alcohol classes, broad-risk alcohol profiles, and cannabis use disorder tend to recruit glutamatergic and reward-circuit plasticity, presynaptic adaptation, AMPK-mTOR nutrient sensing, mitochondrial bioenergetic handling, and selected NAD/sirtuin stress-response branches. Anxiety diverges by loading more strongly on inflammatory-apoptotic signaling, complement/SARM1-linked axonal stress, and mitochondrial NAD-stress programmes. These are predicted-expression association patterns and pathway-level hypotheses, not direct measurements of disease-state gene expression. This framework explains why exposure versus dependence, Class 2 versus Class 3 or Class 4, and cannabis use disorder versus anxiety can show directional oppositions rather than simple severity scaling.

Discussion and conceptual synthesis

**Figure 2:**
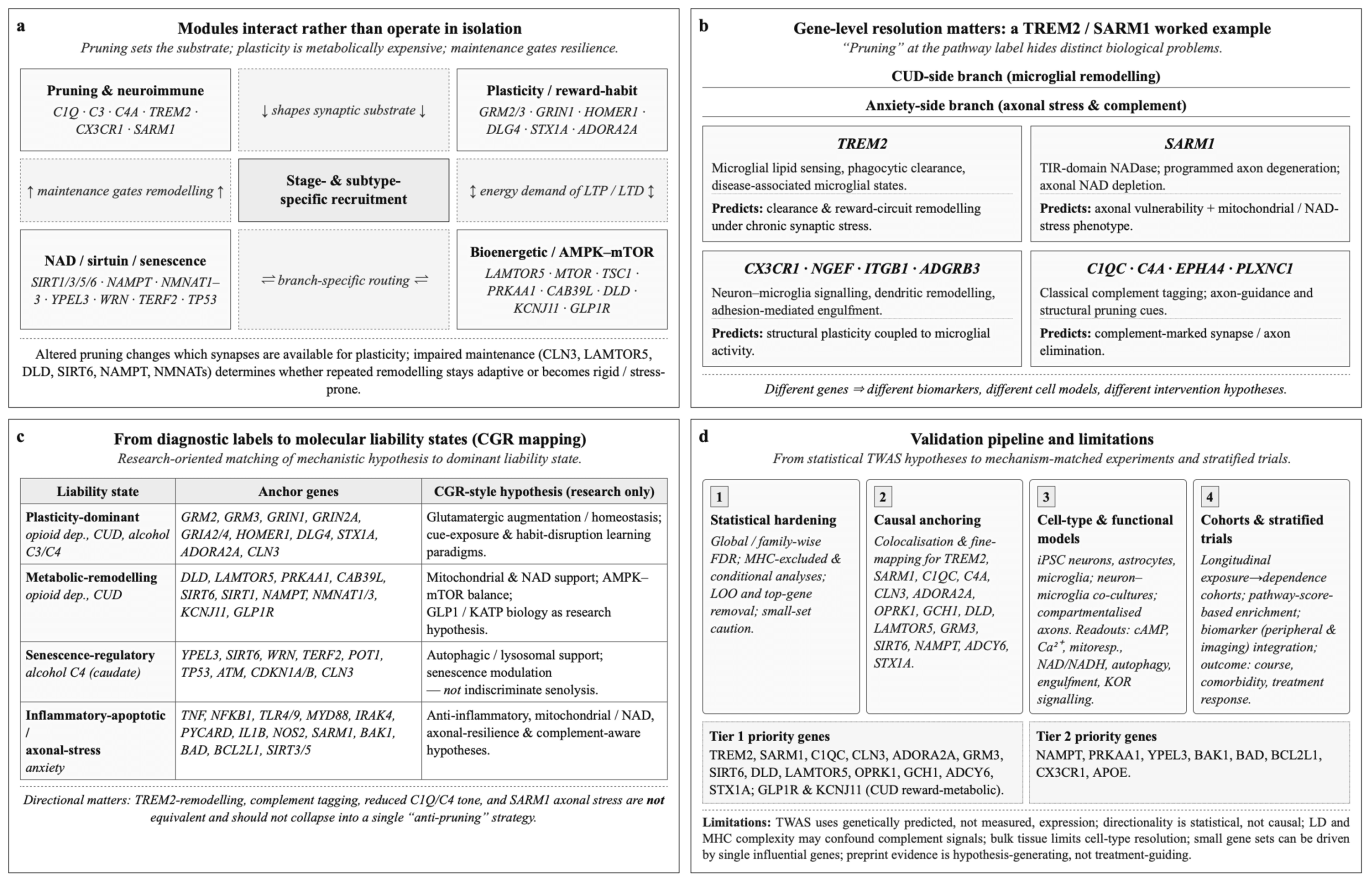
Conceptual synthesis. (a) The four mechanistic modules of the Pruning–Plasticity–Aging architecture interact: pruning sets the synaptic substrate on which plasticity acts; long-term remodelling is metabolically expensive and depends on AMPK–mTOR, mitochondrial flux and NAD/sirtuin routing; maintenance genes (e.g., *CLN3*, *LAMTOR5*, *DLD*, *SIRT6*, *NAMPT*, *NMNAT1–3*) determine whether repeated remodelling stays adaptive or becomes rigid. (b) The TREM2/SARM1 contrast illustrates why gene-level resolution is essential: a CUD-side microglial-remodelling branch (*TREM2*,* CX3CR1*,* NGEF*,* ITGB1*,* ADGRB3*) and an anxiety-side axonal-stress/complement branch (*SARM1*,* C1QC*,* C4A*,* EPHA4*,* PLXNC1*) generate different predictions for biomarkers, cellular models, and interventions despite sharing the broad label “pruning”. (c) Translational mapping shifts from diagnostic labels to dominant molecular liability states (plasticity-dominant, metabolic-remodelling, senescence-regulatory, inflammatory-apoptotic/axonal-stress), each linked to anchor genes and to a CGR-style research hypothesis (glutamatergic, NAD/mitochondrial, autophagic/senescence-modulating, anti-inflammatory/axonal-resilience). Directional caveats are emphasised because not all pruning-related strategies are equivalent. (d) A four-stage validation pipeline — statistical hardening, causal anchoring by colocalisation and fine-mapping, cell-type-resolved functional models, and stratified longitudinal cohorts or trials — operates on Tier 1 and Tier 2 priority genes. Limitations of the underlying TWAS framework are stated explicitly: all statements remain hypothesis-generating until measured expression, proteomic, and functional evidence are added. AMPK-mTOR = AMP-activated protein kinase-mechanistic target of rapamycin; CUD = cannabis use disorder; NAD = nicotinamide adenine dinucleotide; TWAS = transcriptome-wide association study

The main contribution of the Pruning-Plasticity-Aging framework is that it gives a structured explanation for a recurring observation in the Cheung preprints: phenotype decomposition changes the molecular profile (Figure 2). If exposure and dependence are combined, or if alcohol misuse is treated as one trait, the resulting signal may average together opposing biological states. In the opioid analyses, exposure-related liability is more closely tied to altered complement and glial refinement, while dependence progression is more strongly linked to KOR/dynorphin negative affect, pain-stage biology, cAMP and calcium adaptation, mitochondrial metabolism, and mTOR/NAD stress-response branches. In alcohol, Class 2 opposes Class 3 and Class 4 in glutamatergic and plasticity profiles. In cannabis use disorder and anxiety, the same broad domain of pruning biology splits into TREM2-positive microglial remodeling on one side and SARM1/C1QC-positive axonal-stress pruning on the other. These findings are more consistent with a stage- and subtype-sensitive architecture than with one general psychiatric-risk program.

Mechanistically, the modules in the framework are likely to interact rather than operate in isolation. More cautiously, the model hypothesizes interaction among modules, but TWAS alone cannot establish causal ordering. Pruning changes the substrate on which plasticity acts. If complement tagging, TREM2-mediated microglial clearance, CX3CR1 signaling, or SARM1-linked axonal stress is altered, the synapses available for reward learning, extinction, habit formation, and relapse may also change. Similarly, plasticity is metabolically expensive. Long-term synaptic remodeling requires protein synthesis, vesicle cycling, ion pumping, mitochondrial ATP production, NAD-dependent redox handling, and autophagic turnover. Genes such as *CLN3*,* LAMTOR5*,* DLD*,* SIRT6*,* NAMPT*, and* NMNAT*-family members are therefore not peripheral to addiction biology. They may help determine whether repeated reward-circuit remodeling remains adaptive, becomes rigid, or crosses into stress-related vulnerability.

The TREM2/SARM1 contrast is one of the clearest examples of why gene-level resolution matters. If one only says that cannabis use disorder and anxiety involve pruning, the conclusion is too broad to be useful. TREM2 suggests microglial lipid handling, phagocytosis, and remodeling states; SARM1 suggests axonal degeneration and NAD consumption; C1QC suggests complement tagging; EPHA4 and PLXNC1 suggest axon guidance and structural pruning. These are different biological problems. A TREM2-positive cannabis use disorder profile could reflect reward-circuit remodeling and clearance under chronic synaptic stress. A SARM1/C1QC-positive anxiety profile could reflect axonal stress, inflammatory complement activation, and mitochondrial NAD vulnerability. These hypotheses make different predictions for biomarkers, cell models, and interventions.

The branch-specific aging finding is also important. A common mistake would be to summarize the Cheung preprints as showing accelerated aging across substance use and anxiety. That would lose the main insight. The evidence instead suggests that aging-related biology is divided into branches. Alcohol subtypes show caudate stress programs involving *YPEL3*, *SIRT6*, *WRN*, *TERF2*, *TP53*, *ATM*, and *CDKN* genes, pointing toward DNA repair, telomere maintenance, chromatin control, and checkpoint biology. CUD shows SIRT6, SIRT1, NMNAT1, NMNAT3, and metabolic-regulatory signals with lower emphasis on inflammatory SASP-like output. Anxiety shows SARM1, SIRT3, SIRT5, NADK2, NMRK1, inflammatory signaling, and apoptosis-threshold genes. These patterns are best described as branch-specific NAD/sirtuin and stress-response routing [14,15,48].

The translational implications are promising but should remain research-oriented. The framework supports the idea that precision psychiatry should move from broad diagnostic labels toward molecular liability states. Instead of asking only whether a person has opioid use disorder, alcohol use disorder, cannabis use disorder, or anxiety, future studies could ask whether the dominant liability state is pruning-related, plasticity-dominant, metabolic-remodeling, NAD/sirtuin-stress, senescence-regulatory, inflammatory-apoptotic, or axonal-stress related. This does not mean current TWAS findings should guide clinical treatment. It means they can guide trial enrichment, biomarker selection, and functional experiments [60].

Research agenda: non-clinical hypothesis mapping

For non-clinical research hypothesis mapping, the clearest implication is that glutamatergic mechanisms are most biologically justified as experimental targets in plasticity-dominant states. These include opioid dependence progression, cannabis use disorder, and alcohol Class 3 or Class 4 profiles, especially when *GRM2*,* GRM3*,* GRIN1*,* GRIN2A*,* GRIA2*,* GRIA4*,* HOMER1*,* DLG4*,* STX1A*,* ADORA2A*, and* CLN3* are implicated. This is not a recommendation for clinical glutamatergic augmentation. It is a proposal for preclinical prioritization, biomarker development, and prospective stratified testing.

Mitochondrial and NAD-support hypotheses are more relevant to metabolic-remodeling states involving *DLD*,* LAMTOR5*,* PRKAA1*,* CAB39L*,* SIRT6*,* SIRT1*,* NAMPT*,* NMNAT1*, and* NMNAT3*. Senescence modulation and autophagic support are most relevant to *CLN3*,* SIRT6*,* YPEL3*,* WRN*,* TERF2*,* TP53*, and* CDKN*-linked profiles, but this should not be simplified into indiscriminate senolysis. Neuroimmune or pruning-related strategies require directional interpretation, because TREM2-remodeling, complement tagging, reduced C1Q/C4 tone, and SARM1 axonal stress are not equivalent.

Candidate pathway scores could be useful in future cohorts only as research instruments. They are not validated biomarkers and should not be used for diagnosis, prognosis, or treatment selection. A pruning score might combine *C1QA*,* C1QB*,* C1QC*,* C3*,* C4A*,* TREM2*,* CX3CR1*,* APOE*,* SARM1*,* EPHA4*, and* PLXNC1*. A plasticity and glutamate score might combine *GRM2*,* GRM3*,* GRIN1*,* GRIN2A*,* GRIA2*,* GRIA4*,* HOMER1*,* DLG4*,* STX1A*,* VAMP2*,* ADORA2A*,* DRD2*,* PPP1R1B*,* COMT*, and* CLN3*. An opioid progression score might combine *OPRK1*,* GCH1*,* ADCY6*,* ADCY9*,* PDE4B*,* PDE1B*,* PRKAR2A*,* PRKAR2B*,* PPP3R1*,* PPP3CC*,* PLCB3*,* LAMTOR5*,* DLD*,* SIRT6*, and* STX1*A. A metabolic AMPK-mTOR score might combine *LAMTOR5*,* MTOR*,* TSC1*,* RPTOR*,* PRKAA1*,* PRKAB1*,* PRKAG1*,* CAB39L*,* DLD*,* KCNJ11*,* ABCC8*,* GLP1R*, and* PCSK2*. A NAD/sirtuin aging score might combine *NAMPT*,* SIRT1*,* SIRT3*,* SIRT5*,* SIRT6*,* NMNAT1*,* NMNAT2*,* NMNAT3*,* NADK2*,* NMRK1*,* PARP1*,* CD38*,* NT5E*,* YPEL3*,* WRN*,* TERF2*,* POT1*,* TP53*,* ATM*,* CDKN1A*, and* CDKN1B*. An anxiety inflammatory-apoptotic score might combine *TNF*,* NFKB1*,* RELA*,* TLR4*,* TLR9*,* MYD88*,* IRAK4*,* PYCARD*,* IL1B*,* NOS2*,* SARM1*,* BAK1*,* BAD*,* BCL2L1*,* CASP10*,* SIRT3*,* SIRT5*, and* NADK2*.

The most important validation targets are the recurrent genes that combine cross-paper recurrence, mechanistic centrality, and translational relevance. Tier 1 genes include *TREM2*,* SARM1*,* C1QC*,* CLN3*,* ADORA2A*,* GRM3*,* SIRT6*,* DLD*,* LAMTOR5*,* OPRK1*,* GCH1*,* ADCY6*, and* STX1A*, with *GLP1R* and *KCNJ11* also important for the cannabis use disorder reward-metabolic branch. Tier 2 genes include *NAMPT*,* PRKAA1*,* YPEL3*,* BAK1*,* BAD*,* BCL2L1*,* CX3CR1*, and* APOE*. The first validation step should be colocalization and fine-mapping, especially for *TREM2*,* SARM1*,* C1QC*,* C4A*,* CLN3*,* ADORA2A*,* OPRK1*,* GCH1*,* DLD*,* LAMTOR5*,* GRM3*,* SIRT6*,* NAMPT*,* ADCY6*, and* STX1A*. Complement and MHC-region signals require special caution because linkage disequilibrium and structural complexity can make causal interpretation difficult [61].

Functional validation should use models matched to the biology of each module. For opioid dependence progression, iPSC-derived striatal or cortical neurons, astrocytes, and microglia could be exposed to opioid and withdrawal-like stress while perturbing *OPRK1*,* GCH1*,* ADCY6*,* PDE4B*,* LAMTOR5*,* DLD*, and* SIRT6*. Readouts should include cAMP rebound, calcium flux, mitochondrial respiration, NAD and NADH state, glutamate release, dynorphin/KOR signaling, and withdrawal-like stress responses. For alcohol Class 4, neuron-microglia and striatal co-cultures could test *CLN3*, *ADORA2A*, *TREM2*, *C1Q* genes, *SIRT6*, *YPEL3*, and *CAB39L* under ethanol exposure and withdrawal. Readouts should include autophagic flux, lysosomal function, synaptic protein turnover, dendritic spines, microglial engulfment, and mitochondrial stress. For the cannabis use disorder/anxiety contrast, compartmentalized axon systems and neuron-microglia co-cultures could test *TREM2*,* SARM1*,* C1QC*,* GLP1R*,* KCNJ11*,* SIRT3*, and* SIRT6*, with readouts for phagocytosis, axon degeneration, NAD depletion, complement tagging, cytokines, glutamate receptor trafficking, and mitochondrial stress.

There are clear limitations. TWAS estimates genetically predicted expression, not measured expression in patients. A positive TWAS association does not mean a gene is empirically upregulated in the brain during illness, and a negative association does not mean it is downregulated. Directionality is statistical, not causal. Several high-priority findings may reflect linkage disequilibrium with nearby causal genes unless colocalization supports the same variant driving both gene expression and phenotype association. Bulk brain TWAS cannot fully resolve whether signals arise from neurons, astrocytes, microglia, oligodendrocytes, endothelial cells, or mixed cell populations. Small gene sets, such as some *DREAM*, pharmacokinetic, *GLP1*, or *BCL-2*-related sets, may be driven by a few influential genes. Finally, because the Cheung papers are preprints and hypothesis-generating syntheses, the framework should be treated as a map for future work rather than as a settled mechanism.

Additional limitations follow from the narrative design of this review. The supporting literature was not selected through a reproducible systematic search, no PRISMA flow diagram was generated, no formal risk-of-bias instrument was applied, and no new quantitative synthesis was performed. The review therefore cannot estimate pooled effects, between-study heterogeneity, publication bias, or statistical robustness across all available TWAS or omics studies. A future quantitative version should include explicit search terms, databases, date limits, inclusion and exclusion criteria, a source-selection flow diagram, formal appraisal of preprints and peer-reviewed studies, and independent statistical or genetic-epidemiology review.

The overall evidence is strongest for the claim that phenotype decomposition changes transcriptomic architecture. It is also strong for opioid exposure versus dependence opposition, alcohol Class 2 versus Class 3 or Class 4 plasticity inversion, and cannabis use disorder versus anxiety pruning divergence. The most novel pieces are the TREM2/SARM1 cross-disorder contrast and the branch-specific aging model. The most immediate impact is likely to be in mechanistic prioritization, biomarker design, and trial enrichment rather than clinical decision-making. In practical terms, the framework is ready to guide experiments, but not yet ready to guide treatment selection.

The five Cheung TWAS preprints and their synthesis support a cross-disorder Pruning-Plasticity-Aging architecture for substance-use and anxiety liability. The central advance is not the discovery that addiction involves glutamate, or that anxiety involves inflammation, or that aging biology overlaps with psychiatric disease. Those ideas already have strong foundations. The advance is the stage- and subtype-sensitive organization of these modules into a coherent model in which opposing transcriptomic profiles can emerge from different phenotype definitions.

## Conclusions

Opioid exposure and dependence progression appear molecularly distinct. Exposure-related liability is more closely linked to altered complement, pruning, and glial-synaptic refinement, while dependence progression is more closely linked to KOR/dynorphin negative affect, cAMP/calcium adaptation, pain-stage biology, mitochondrial flux, AMPK-mTOR signaling, and SIRT6/NAD-related stress routing. Alcohol misuse is not one transcriptomic state: Class 2 shows plasticity profiles that oppose heavier and broader-risk classes, while Class 4 adds CLN3, ADORA2A, TREM2, complement, and caudate senescence-like stress biology. Cannabis use disorder and anxiety share some glial-metabolic background, but diverge sharply in pruning biology, with cannabis use disorder showing TREM2-linked remodeling and anxiety showing SARM1/C1QC-linked axonal-stress and complement biology. Aging-related signals are branch-specific, not globally accelerated. This framework encourages a shift from broad diagnostic categories to molecular liability states. Future work should test whether pruning, plasticity, metabolic remodeling, NAD/sirtuin stress, senescence-regulatory, and inflammatory-apoptotic profiles can predict course, comorbidity, biomarker status, or treatment response. The most urgent next steps are colocalization, fine-mapping, MHC-sensitive complement analyses, cell-type-specific TWAS, measured expression and proteomic validation, iPSC and co-culture experiments, and longitudinal cohorts with careful exposure, dependence, withdrawal, pain, anxiety, and subtype phenotyping. At present, the framework should be used for mechanistic prioritization and study design only. It should not be used for clinical diagnosis, prognosis, medication selection, or patient-level treatment matching. If validated, the Pruning-Plasticity-Aging model could provide a useful bridge between psychiatric genetics, cellular maintenance biology, and precision intervention design.
